# Left atrial appendage orifice area and morphology is closely associated with flow velocity in patients with nonvalvular atrial fibrillation

**DOI:** 10.1186/s12872-021-02242-9

**Published:** 2021-09-16

**Authors:** Lei Chen, Changjiang Xu, Wensu Chen, Chaoqun Zhang

**Affiliations:** 1 Department of Cardiology, The Affiliated Hospital of Xuzhou Medical University, Xuzhou, 221002 Jiangsu China; 2 Department of Cardiology, The Affiliated Suqian First People’s Hospital of Nanjing Medical University, Suqian, 223800 Jiangsu China

**Keywords:** Atrial fibrillation, Left atrial appendage orifice area, Left atrial appendage mechanical function

## Abstract

**Background:**

Thromboembolic events are the most serious complication of atrial fibrillation (AF), and the left atrial appendage (LAA) is the most important site of thrombosis in patients with AF. During the period of COVID-19, a non-invasive left atrial appendage detection method is particularly important in order to reduce the exposure of the virus. This study used CT three-dimensional reconstruction methods to explore the relationship between LAA morphology, LAA orifice area and its mechanical function in patients with non-valvular atrial fibrillation (NVAF).

**Methods:**

A total of 81 consecutive patients with NVAF (36 cases of paroxysmal atrial fibrillation and 45 cases of persistent atrial fibrillation) who were planned to undergo catheter radiofrequency ablation were enrolled. All patients were examined by transthoracic echocardiography (TTE), TEE, and computed tomography angiography (CTA) before surgery. The LAA orifice area was obtained according to the images of CTA. According to the left atrial appendage morphology, it was divided into chicken wing type and non-chicken wing type. At the same time, TEE was performed to determine left atrial appendage flow velocity (LAAFV), and the relationship between the left atrial appendage orifice area and LAAFV was analyzed.

**Results:**

The LAAFV in Non-chicken wing group was lower than that in Chicken wing group (36.2 ± 15.0 cm/s vs. 49.1 ± 22.0 cm/s, *p*-value < 0.05). In the subgroup analysis, the LAAFV in Non-chicken wing group was lower than that in Chicken wing group in the paroxysmal AF (44.0 ± 14.3 cm/s vs. 60.2 ± 22.8 cm/s, *p*-value < 0.05). In the persistent AF, similar results were observed (29.7 ± 12.4 cm/s vs. 40.8 ± 17.7 cm/s, *p*-value < 0.05). The LAAFV in persistent AF group was lower than that in paroxysmal AF group (34.6 ± 15.8 cm/s vs. 49.9 ± 20.0 cm/s, *p*-value < 0.001). The LAAFV was negatively correlated with left atrial dimension (R = − 0.451, *p*-value < 0.001), LAA orifice area (R= − 0.438, *p*-value < 0.001) and left ventricular mass index (LVMI) (R= − 0.624, *p*-value < 0.001), while it was positively correlated with LVEF (R = 0.271, *p*-value = 0.014). Multiple linear regression analysis showed that LAA morphology (β = − 0.335, *p*-value < 0.001), LAA orifice area (β = −  0.185, *p*-value = 0.033), AF type (β = − 0.167, *p*-value = 0.043) and LVMI (β = − 0.465, *p*-value < 0.001) were independent factors of LAAFV.

**Conclusions:**

The LAA orifice area is closely related to the mechanical function of the LAA in patients with NVAF. The larger LAA orifice area and LVMI, Non-chicken wing LAA and persistent AF are independent predictors of decreased mechanical function of LAA, and these parameters might be helpful for better management of LA thrombosis.

## Background

Atrial fibrillation (AF) is currently the most common arrhythmia encountered in clinical practice. Epidemiological surveys show that the incidence of AF in the population is approximately 0.4–1%. With the aging of the global population, the incidence rate is gradually increasing. It is estimated that, by 2035, the incidence of AF will double the current incidence [1]. AF poses a huge threat and can cause serious damage to the life and health of patients, greatly increasing the risk of ischemic stroke, systemic artery embolism, heart failure, and other diseases, as well as having high disability and fatality rates [2–5]. Thromboembolic events are the most serious complication of AF. Studies have shown that the left atrial appendage is the most important site of thrombosis in patients with AF, and nonvalvular atrial fibrillation (NVAF) thrombi are almost all located in the left atrial appendage [6, 7]. Previous studies have shown that left atrial appendage flow velocity (LAAFV) is closely related to the formation of left atrial mural thrombus and spontaneous imaging [8]. Transesophageal echocardiography (TEE) is currently the most widely used examination method to assess left atrial appendage function and thrombosis [9], but this examination is semi-invasive. The examination process is relatively painful, and there are many contraindications. In addition, the coronavirus disease-19 (COVID-19) has spread globally and is still difficult to control, during the period of COVID-19, the guidelines recommend that it is best to use non-invasive imaging methods to reduce the risk of virus exposure [10–12]. With the development of CT and 3D reconstruction technologies, 3D CT reconstruction has become a simple and reliable way to understand the structure and morphology of the left atrial appendage [7, 8]. At present, there are few relevant studies on the left atrial appendage (LAA) morphology or orifice area on its mechanical function, and there are still controversies. This study investigates the relationship between LAA morphology, LAA orifice area and its mechanical function in patients with NVAF and attempts to identify an effective factor that can predict the reduction in mechanical function of LAA.

## Methods

### Research objects

A retrospective analysis of patients with NVAF who were hospitalized in the Department of Cardiology, Affiliated Hospital of Xuzhou Medical University from November 2016 to November 2020. All patients received standardized drug treatment and management. Inclusion criteria included AF was diagnosed by ECG or 24-h Holter, all patients received TTE, TEE, and CTA examinations, and the interval between TEE and CTA examinations was within 48 h, patients with paroxysmal AF (PaAF) show sinus rhythm during TEE examination, and those with persistent AF (PeAF) show an AF rhythm. Exclusion criteria included incomplete clinical data, complications with valvular heart disease (moderate to severe mitral valve stenosis), patent foramen ovale, artificial valve replacement, poor CT image quality and complete LAA data that could not be obtained, severe liver and kidney dysfunction, patients with thyroid disease or multiple organ dysfunction, etc. Finally, a total of 81 patients were selected, including 36 cases (44.4%) with PaAF and 45 cases (55.6%) with PeAF, with an average age of 61.8 ± 8.8 years. The basic clinical data of the patients are shown in Table 1.

**Table 1:** Clinical data of the study population

Variables	All population(*n* = 81)	PaAF (*n* = 36)	PeAF (*n* = 45)	*P*-value
Age (years)	61.8 ± 8.8	63.2 ± 8.8	60.7 ± 8.8	0.198
Male (*n*, %)	57 (70.4)	26 (72.2)	31 (68.9)	0.744
BMI (kg/m²)	25.6 ± 3.0	25.5 ± 3.1	25.7 ± 3.0	0.722
Hypertension (*n*,%)	30 (37.0)	12 (33.3)	18 (40.0)	0.537
Diabetes (*n*,%)	11 (13.6)	5 (13.9)	6 (13.3)	1.000
CAD (*n*,%)	27 (33.3)	13 (36.1)	14 (31.1)	0.635
Past Stroke (*n*,%)	16 (19.8)	5 (13.9)	11 (24.4)	0.236
CHF (*n*,%)	11 (13.6)	6 (16.7)	5 (11.1)	0.690
Smokers (*n*,%)	24 (29.6)	13 (36.1)	11 (24.4)	0.253
CHA_2_DS_2_-VAScscores	2.1 ± 1.6	2.1 ± 1.6	2.2 ± 1.6	0.730

*BMI* Body mass index, *CAD* coronary artery disease, *CHF* congestive heart failure

### Diagnostic criteria for atrial fibrillation

**Fig. 1:**
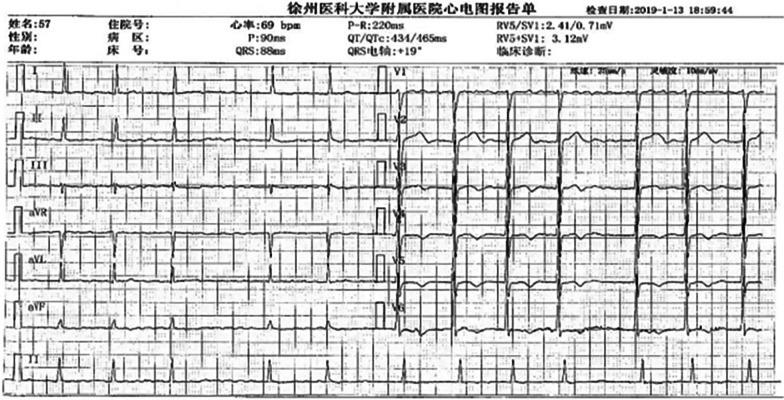
ECG of atrial fibrillation

The electrocardiogram or 24-h Holter electrocardiogram showed the disappearance of P waves and replaced them with f waves of different sizes, shapes, and amplitudes. The frequency of the f wave is 350 to 600 times/min, and the R-R interval is absolutely unequal (Fig. 1). According to the duration of AF, it is divided into PaAF and PeAF. PaAF is defined as AF that terminates within 7 days, and PeAF is defined as AF that lasts more than 7 days [13].

### Transthoracic echocardiography (TTE) examination

A Philips EPIQ 7c ultrasonic diagnostic apparatus, S5-1 probe, and probe with a frequency 1–5 MHz were used. The patient was placed in the left decubitus position, and the patient’s left atrium anteroposterior diameter, left ventricular end-diastolic diameter, left ventricular ejection fraction (measured by biplane Simpson method), left ventricular posterior wall thickness, ventricular septal thickness and other parameters were recorded in detail. The Devereux formula [14] was used to calculate the left ventricular mass index (LVMI), as follows: LVMI (g/m^2^) = LVM (calculated by 0.8 × 1.04 [(IVSd + PWTd + LVDd)^3^ − LVDd^3^] + 0.6)/BSA (calculated by 0.006 × height (cm) + 0.013 × body weight (kg) − 0.153).

### TEE examination and the determination of LAAFV

**Fig. 2:**
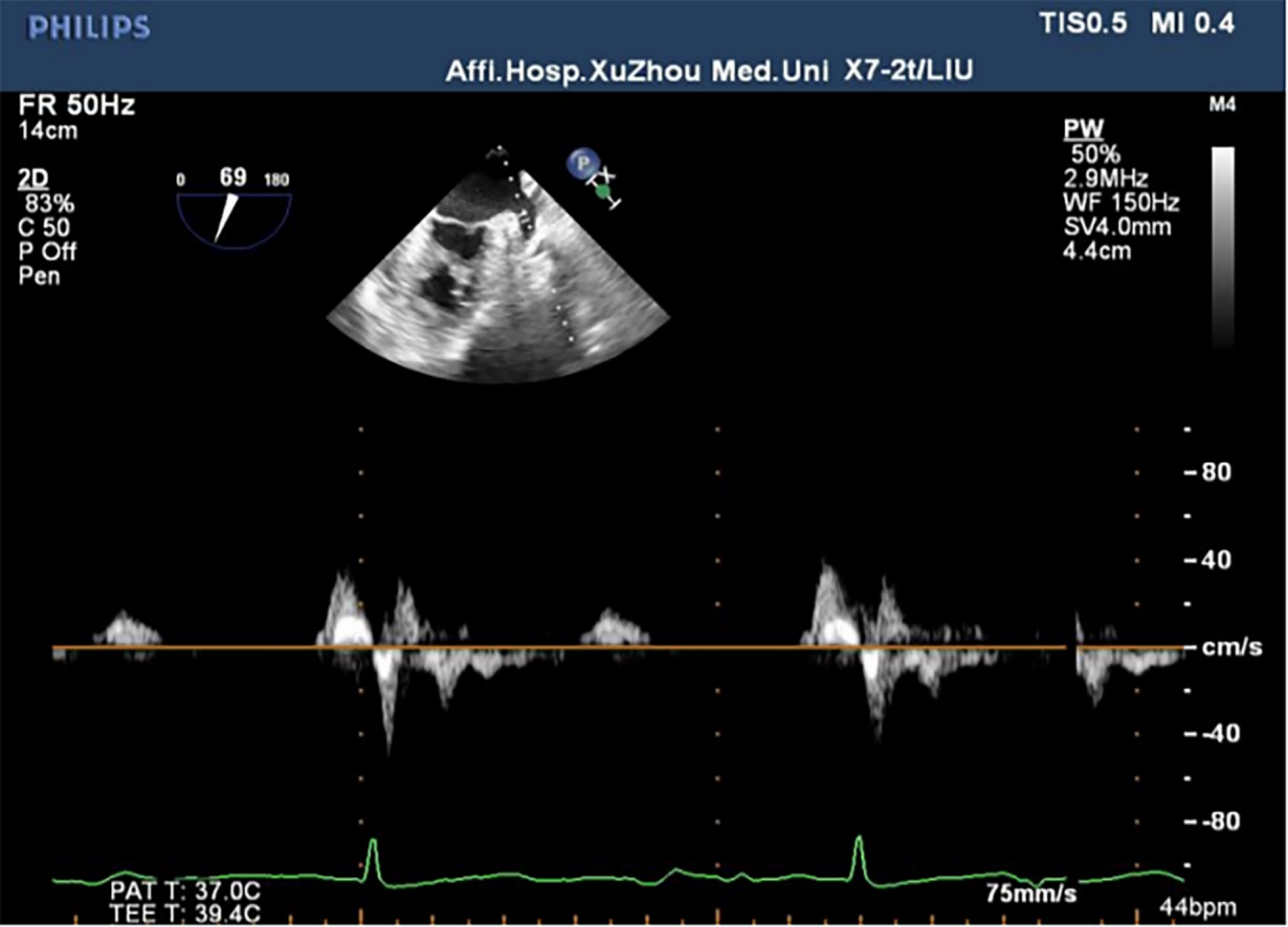
The blood flow pattern of the left atrial appendage in sinus rhythm

**Fig. 3:**
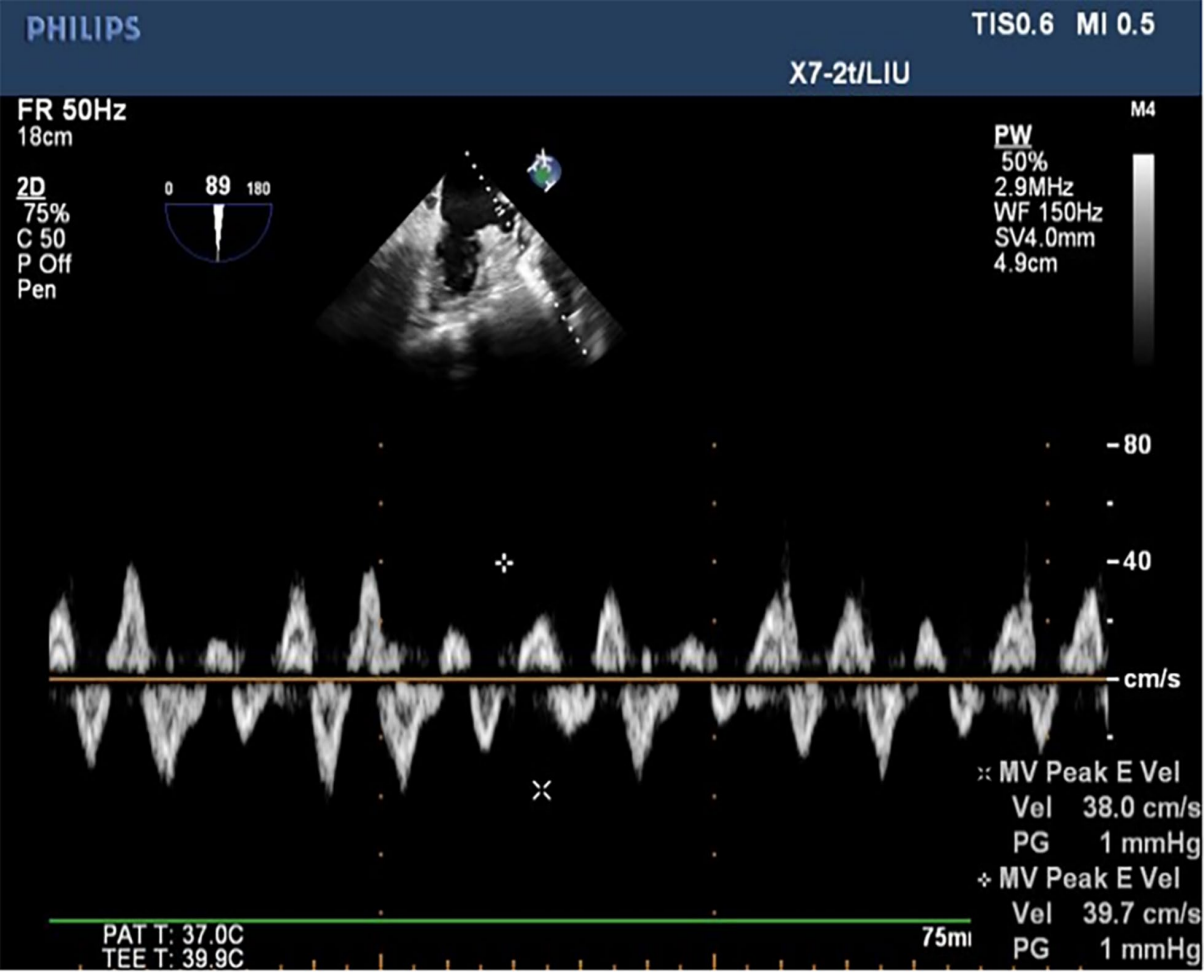
The blood flow pattern of the left atrial appendage in AF rhythm

A Philips iE33 color Doppler ultrasound diagnostic apparatus and a X7-2t transesophageal matrix real-time 3D probe with a frequency of 2–7 MHz were used. Before the examination, patients fasted for 6–8 h. They were connected to the ECG synchronization recording, the pulsed wave Doppler sampling volume was placed within one-third of the proximal opening of the LAA, and the LAA blood flow spectrum was obtained. The blood flow spectrum of the LAA is a regular two-way wave in sinus rhythm and an irregular sawtooth waveform in AF. Record the peak value of the positive wave (maximum emptying speed of the left atrial appendage) within 3 cardiac cycles, and take the average value as left atrial appendage flow velocity (LAAFV) [15] (Figs. 2 and 3).

### Computed tomography angiography (CTA) and 3D reconstruction

The CT imaging data were acquired by the German Siemens dual-source CT machine (SOMATOM Definition, SIEMENS Germany). Iodomidol (60–80 ml) was injected into the cubital vein at a flow rate of 5 ml/s, and then 50 ml of normal saline was injected at a rate of 5 ml/s. Contrast agent tracking technology triggered enhanced scanning. The following scan parameters were used—trigger plane: ascending aorta root level, trigger threshold: 90–100 Hu, start scanning after 6 s delay, scanning time 5–12 s, scanning range: 1 cm below tracheal carina to 1.5 cm lower edge of the heart, detector width 2.0 mm × 32.0 mm × 0.6 mm, layer thickness 2.0 mm × 64.0 mm × 0.6 mm, frame rotation time 330 ms, heart rate dependent pitch 0.2–0.5, tube current 400 mA, and voltage 120 kV.

### Measurement of LAA volume and its morphology

**Fig. 4:**
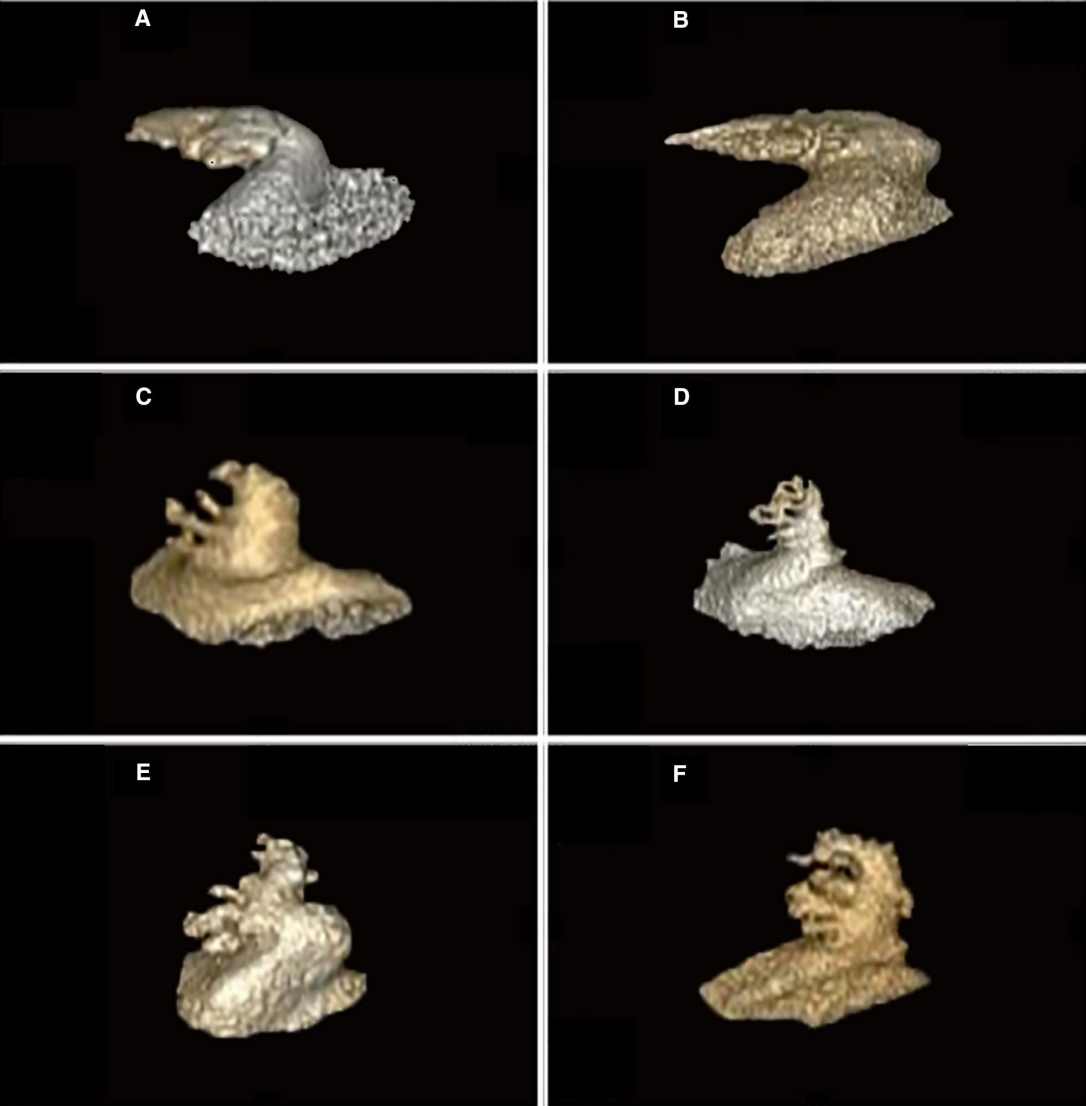
The shape of the LAA. **A**, **B** Chicken-wing. **C**–**F** Non-chicken wing

**Fig. 5:**
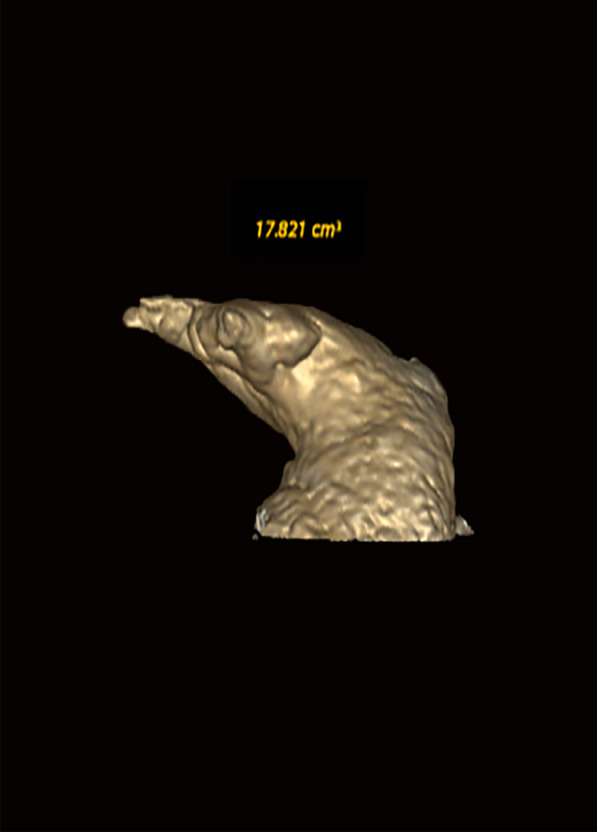
Left atrial appendage volume

The GE AW4.6 Workstation was used to perform 3D reconstruction of the original CT images to obtain 3D images of the LAA and left atrium (LA). Then, a cutting tool was used to separate the LAA from the LA to obtain the LAA volume. According to the morphological characteristics of the LAA, it can be divided into chicken wing (there is an obvious fold at the proximal or middle part of the main lobe of the left atrial appendage) and non-chicken wing (other forms beside chicken wings) (Figs. 4 and 5).

### Measurement of the LAA orifice area

**Fig. 6:**
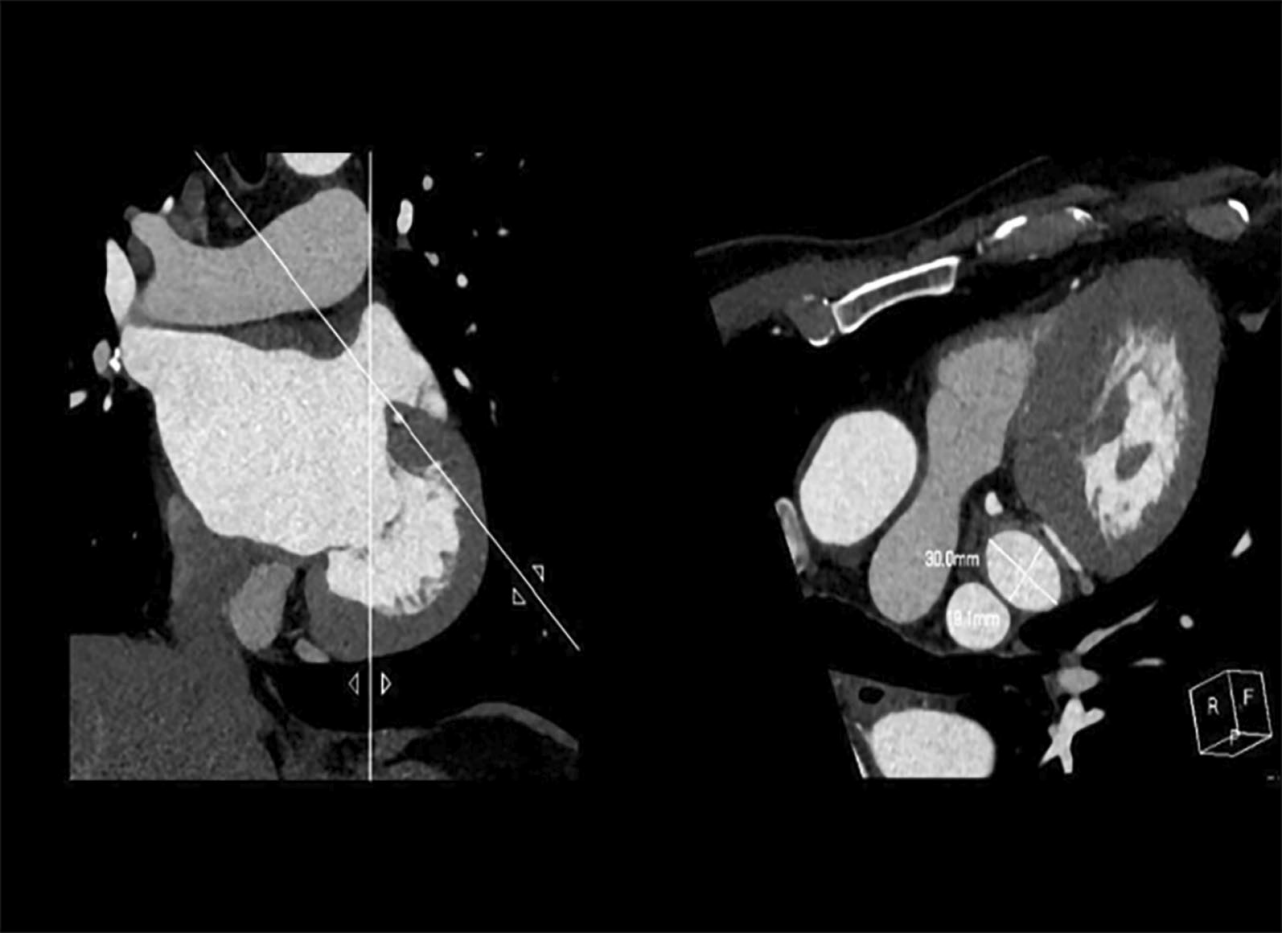
Measurement of the length and short diameter of the left atrial appendage orifice

The long diameter (D1) and short diameter (D2) of the LAA orifice were measured by a Philips IntelliSpace Portal workstation. The LAA orifice was manually cross-sectioned from the multiplanar reconstruction image, and the orifice area was determined by its narrowest part. By creating a plane perpendicular to the axis of the left atrial ear neck, a cross-sectional view of the LAA is generated (Fig. 6). The formula 0.785*D1*D2 was used to calculate the LAA orifice area [16].

### Statistical analysis

SPSS 22.0 software was used for statistical analysis. Measurement data are expressed as the mean ± standard deviation, and independent sample t-test was used for comparison between groups. Count data are expressed by the number of cases and percentage (%), and the χ^2^ test or Fisher’s exact probability method was used for comparison between groups. T-test or simple linear regression analysis was used to determine each parameter that may affect LAAFV. Then, the findings were incorporated into multiple linear regression analysis to evaluate the independent predictors that determine LAAFV. *P*-value < 0.05 was considered statistically significant.

## Results

### Comparison of baseline data

As shown in Table 1. There were no significant differences in age, sex, BMI, hypertension, diabetes, coronary artery disease, past stroke, congestive heart failure, smokers and CHA2DS2-VASc scores between PeAF group and PaAF group (*p*-value > 0.05).

### Comparison of ultrasound and 3D CT reconstruction of left atrial appendage data

There were no significant differences in LVEDD, LVST, LVPWT, LVEF, E/e’ between PeAF group and PaAF group (*p*-value > 0.05). The LAD and LAA orifice area in PeAF group were larger than that in PaAF group (*p*-value < 0.05), and LAAFV was lower than that in PaAF group (*p*-value < 0.001). There was no significant difference in LAA morphology and LAA volume between the two groups through CT (*p*-value > 0.05). However, the LVMI in PeAF group was larger than that in PaAF group (*p*-value < 0.001) (Table 2).

**Table 2:** Ultrasound and 3D CT reconstruction of left atrial appendage data

Variables	All population(*n* = 81)	PaAF (*n* = 36)	PeAF (*n* = 45)	*P*-value
LAD (mm)	41.9 ± 5.3	40.3 ± 4.3	43.2 ± 5.7	0.016
LVEDD (mm)	49.7 ± 5.2	49.1 ± 3.5	50.2 ± 6.2	0.344
IVST (mm)	9.5 ± 1.4	9.4 ± 1.5	9.5 ± 1.3	0.694
LVPWT (mm)	9.2 ± 1.2	9.0 ± 1.0	9.4 ± 1.4	0.146
LVEF (%)	61.6 ± 9.8	63.6 ± 8.4	59.9 ± 10.7	0.101
E/e’	8.0 ± 2.8	7.5 ± 2.4	8.5 ± 3.1	0.105
LAAFV (cm/s)	41.8 ± 19.3	50.8 ± 19.8	34.6 ± 15.8	< 0.001
Non-chicken wing (*n*,%)	46(56.8)	21(58.3)	25(55.6)	0.802
LAA orifice area (cm²)	4.7 ± 1.5	4.2 ± 1.6	5.1 ± 1.4	0.007
LAA volume (ml)	11.5 ± 5.1	10.8 ± 5.0	12.1 ± 5.2	0.237
LVMI (g/m^2^)	101.35 ± 31.91	88.13 ± 19.28	111.92 ± 36.03	< 0.001

*LAD* left atrial dimension, *LVEDD* left ventricular end diastolic dimension, *IVST* interventricular septal thickness, *LVPWT* left ventricular posterior wall thickness, *LVEF* left ventricular ejection fraction, *LVMI* left ventricular mass index

### Analysis of relevant parameters of LAAFV by t-test.

**Fig. 7:**
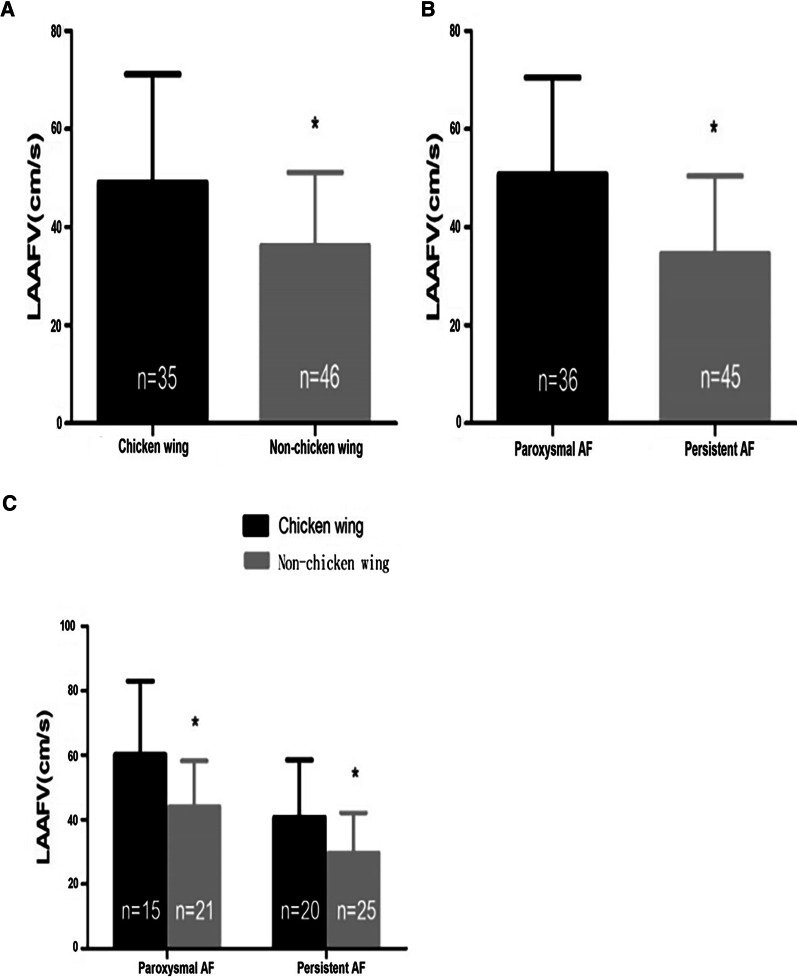
Analysis of relevant parameters of LAAFV by t test. **A** Comparison of LAAFV between Chicken wing group and Non-chicken wing group. *p-value < 0.05: Comparison between Chicken wing and Non-chicken wing. **B** Comparison of LAAFV between PeAF and PaAF. *p-value < 0.05: Comparison between PeAF group and PaAF. **C** Comparison of LAAFV between Chicken wing group and Non-chicken wing group in different types of AF. *p-value < 0.05: Comparison between Chicken wing and Non-chicken wing

Among all the enrolled patients, the LAAFV in Non-chicken wing group was lower compared with that in Chicken wing group (36.2 ± 15.0 cm/s vs. 49.1 ± 22.0 cm/s, *p*-value < 0.05) (Fig. 7A). Among different types of AF, the LAAFV in PeAF group was lower compared with that in PaAF group (34.6 ± 15.8 cm/s vs. 49.9 ± 20.0 cm/s, *p*-value < 0.001) (Fig. 7B). In the subgroup analysis, the LAAFV in Non-chicken wing group was lower than that in Chicken wing group in PaAF (44.0 ± 14.3 cm/s vs. 60.2 ± 22.8 cm/s, *p*-value < 0.05); The LAAFV in Non-chicken wing group was also lower than that in Chicken wing group in PeAF (29.7 ± 12.4 cm/s vs. 40.8 ± 17.7 cm/s, *p*-value < 0.05) (Fig. 7 C).

### Analysis of relevant parameters of LAAFV by simple linear regression

**Fig. 8:**
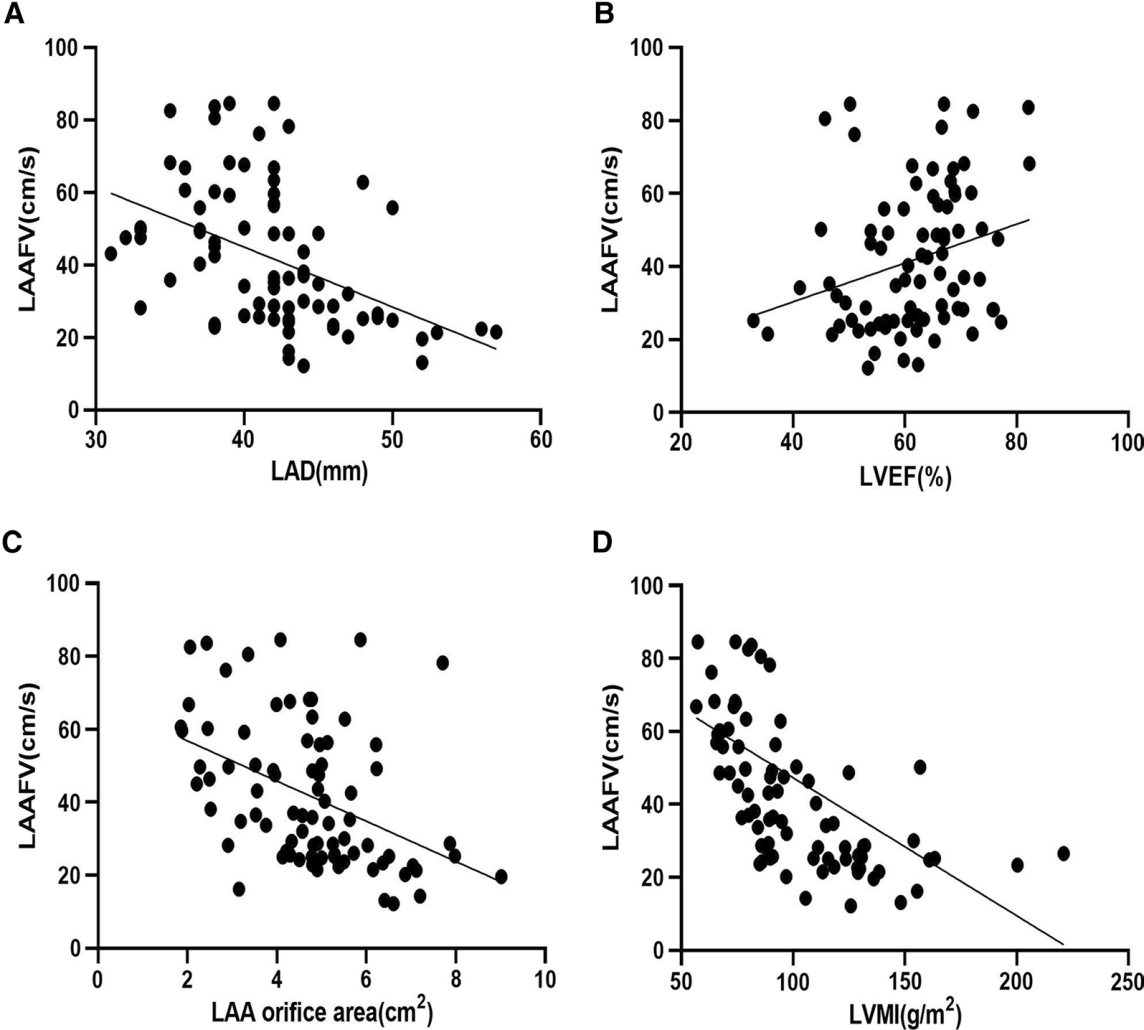
Analysis of relevant parameters of LAAFV by simple linear regression. **A** Comparison between LAAFV and LAD (Y = 110.940-1.65X). **B** Comparison between LAAFV and LVEF (Y = 9.075 + 0.532X). **C** Comparison between LAAFV and LAA orifice area (Y = 67.858-5.506X). **D** Comparison between LAAFV and LVMI (Y = 80.120-0.378X)

The LAAFV was negatively correlated with the LAD (R = − 0.451, *p*-value < 0.001) (Fig. 8A), the LAA orifice area (R = − 0.438, *p*-value < 0.001) (Fig. 8C) and LVMI (R = − 0.624, *p*-value < 0.001) (Fig. 8D). While it was positively correlated with the LVEF (R = 0.271, *p*-value = 0.014) (Fig. 8B).

### Multiple linear regression analysis of LAAFV predictors.

The statistically significant variables were included in the multiple linear regression analysis. The multiple linear regression analysis showed that LAA morphology (β = − 0.335, *p*-value < 0.001), LAA orifice area (β = − 0.185, *p*-value = 0.033), AF type (paroxysmal vs. persistent) (β = − 0.167, *p*-value = 0.043) and LVMI (β = − 0.465, *p*-value < 0.001) were independent factors of LAAFV (Table 3).

**Table 3:** Multiple linear regression analysis of LAAFV predictors

Variables	B	SE	β	T	*P*-value
Constant	114.633	17.957		6.384	0.000
AF type (paroxysmal vs. persistent)	− 6.462	3.136	− 0.167	− 2.060	0.043
LAD	− 0.258	0.333	− 0.071	− 0.775	0.441
LVEF	0.128	0.153	0.065	0.840	0.404
LAA orifice area	− 2.324	1.068	− 0.185	− 2.177	0.033
LAA morphology (Non-chicken wing vs. chicken wing)	− 12.974	2.858	− 0.335	− 4.540	< 0.001
LVMI	− 0.281	0.053	− 0.465	− 5.326	< 0.001
R² adjusted	0.569				
R²	0.602				

*LAD* left atrial dimension, *LVEF* left ventricular ejection fraction, *LVMI* left ventricular mass index

## Discussion

The LAA is a finger-like extension originating from the main body of LA [17]. It has active systolic and diastolic functions, and its mechanical dysfunction may lead to blood flow stagnation and thrombosis [18]. At present, the LAAFV measured by TEE is the most commonly used method to evaluate the mechanical function of the LAA. A large number of studies have shown that the risk of thrombosis is steadily increased with the decrease of LAAFV [19–21]. Handke et al. [8] conducted a TEE study on 500 patients with cerebral ischemia and found that the measurement of the LAAFV may be an important quantitative substitute parameter for evaluating the risk of left atrial thromboembolism. However, TEE is semi-invasive and requires higher personal experience of the operator and may cause complications, such as bleeding and perforation [22]. There are certain checkups, such as combined esophageal stenosis. Therefore, looking for noninvasive examination indicators that can effectively predict the mechanical function of the LAA has important clinical significance. Especially during the period of COVID-19.

At present, research on the anatomy of the LAA is still rare, and there is no uniform standard for the classification of the LAA. With the rapid development of multi-slice spiral CT and 3D reconstruction technology, it is possible to observe heart anatomy and vascular structure in detail. According to CT or MRI images of the heart, Wang [23] et al. divided LAA into four categories: chicken wing type, weathercock type, cauliflower type and cactus type. Among them, the chicken wing type is the most common form. However, due to the complex structure of LAA, sometimes it will show different morphological characteristics when viewed from different angles.It has been reported in the literature that this classification method is subjective [24]. Therefore, we refer to the relevant literature to divide LAA morphology into chicken wing type and non-chicken wing type. This classification can significantly reduce subjectivity. Previous studies have shown that LAA morphology is related to the LAAFV [25, 26]. Fukushima et al. [26] reported that compared with the cactus type and cauliflower type LAA, the chicken-wing type LAA had a significantly higher flow velocity, but there was no statistical difference compared with the weathercock type. However, the study only included patients with PaAF. Our study found that whether it is in PaAF group or PeAF group, the LAA morphology is closely related to its mechanical function. The LAAFV of chicken-wing AF is higher than that of non-chicken wing AF. The possible mechanisms are as follows: First, chicken-wing patients may have greater muscle mass to contract the LAA [27]. Second, high left atrial pressure or low left atrial compliance may change LAA morphology[25].

Compared with the direct measurement after CT three-dimensional reconstruction of the LAA, the measurement method used in this study judges that the LAA orifice is relatively less subjective and the measurement repeatability is good [23]. At present, the research on LAA orifice area and the mechanical function of LAA in patients with AF has not been reported in the literature. Our study showed that the increase of LAA orifice area in patients with NVAF is closely related to the decrease of LAAFV. Agmon et al. [28] reported that the emptying speed of the LAA of the normal population was negatively correlated with the diameter of the LAA orifice measured by TEE (r=-0.29, *p*-value = 0.002). According to the continuity equation, as the cross-sectional area increases, the flow velocity becomes slower. In the case of constant flow, as the cross-sectional area decreases, the flow velocity becomes faster. Our data showed that the relationship between the LAA orifice area and its flow velocity in NVAF patients also conforms to the same law, so LAA orifice area is an important factor in determining LAAFV. Studies have shown that LAAFV was significantly negatively correlated with the LAD [29, 30]. Similar to the results of these studies, we found that LAD is negatively correlated with LAAFV. The reasons may be as follows: With the progress of AF, LA gradually undergoes structural remodeling, which in turn leads to an increase in the inner diameter and pressure of LA. The compliance of LAA is greater than that of the LA and can regulate left atrial pressure. However, the increase in left atrial pressure can lead to an increase in the afterload of LAA and lead to the decrease of LAAFV [31]. This can also explain the view that some scholars believe that LAD can predict the risk of stroke[32]. However, similar to the study of Harada M et al. [33], the multiple linear regression analysis did not prove that LAD is an independent factor of the LAAFV. Therefore, this needs more research to confirm.

The current guidelines recommend that AF be divided into five categories: primary AF, PaAF, PeAF, long-term PeAF and permanent AF [13]. Petersen et al. [34] found that from PaAF group, to PeAF, and then to long-term PeAF group, the LAAFV gradually decreased (51.4 ± 25.1 cm/s vs. 40.9 ± 16.3 cm / s vs. 29.7 ± 15.1 cm/s, *p*-value < 0.001); Multiple linear regression analysis found that AF type was an independent predictor of LAAFV. It is consistent with our research results, and the reasons may be as follows: First, during the TEE and TTE examinations in this study, patients in the PaAF group were required to maintain sinus rhythm. When the heart rhythm is AF, the emptying time of LA and LAA is shortened, so the volume of LA is relatively increased. The rapid and irregular electrical activity will weaken the contractility of LAA, resulting in the decrease of LAAFV. Secondly, PeAF usually has a longer course than PaAF, the electrical remodeling and fibrosis of LA are more serious, which is more likely to cause an increase in the load of the LAA and cause systolic dysfunction, leading to the LAAFV was lower in PeAF group than that in PaAF group. LVEF is a common indicator reflecting left ventricular function. Our study found that LAAFV is positively correlated with LVEF. However, the multiple linear regression analysis did not prove that LVEF is an independent predictor of the LAAFV. This is similar to the study of Kishima et al. [25]. The reason may be that the population included in our study is mainly NVAF patients with normal LVEF. So far, studys on the relationship between LVMI and LAA are rare, and there seems to be some controversy [33, 35]. Our study showed that LAAFV was negatively correlated with LVMI, and the multiple linear regression analysis proved that LVMI is an independent factor of the LAAFV, which is similar to the study of Harada M et al. [33].

At present, the CHA2DS2-VASc score is the most commonly used index for clinical assessment of stroke risk stratification in NVAF patients, and is used to guide anticoagulation therapy [36]. The 2016 ESC guidelines recommended that patients with CHA2DS2-VASc score ≥ 1 can choose anticoagulation therapy [37]. However, previous studies have found that patients with a score of 0 are still at risk of ischemic stroke. Gage et al. found that NVAF patients with a CHA2DS2-VASc score of 0 have an annual stroke risk as high as 1.9% [38]. Moreover, the score mainly focuses on clinical indicators, and does not pay attention to the influence of heart structure and function on thrombosis. Therefore, the scoring system is not sufficient to comprehensively assess the risk of stroke in patients. Our study showed that non-chicken wing LAA in patients with NVAF can cause the decrease of LAAFV, which may increase the risk of thrombosis. Our research on the morphology and mechanical function of LAA may provide additional clinical significance for stroke risk stratification in NVAF patients. However, this study still has some limitations. First, this study is a retrospective, single-center, small-sample study. As a retrospective study, it was regretted that we did not discuss LA volume in this study. It is hoped that there will be a larger cohort for further prospective studies in the future. Second, there is still no uniform standardthe for the LAA morphology. Some clinical studies divide it into four types, this study only classifies it into two types. This classification can minimize the difference between observers, but it may not be precise enough.

## Conclusions

The LAA orifice area is closely related to the mechanical function of the LAA in patients with NVAF. The larger LAA orifice area and LVMI, Non-chicken wing LAA and PeAF are independent factors of decreased mechanical function of LAA, and these parameters might be helpful for better management of LA thrombosis.

## Data Availability

The datasets used and/or analyzed during the present study are available from the corresponding author on reasonable request.

## Abbreviations

AF: Atrial fibrillation
BMI: Body mass index
CHF: Congestive heart failure
CTA: Computed tomography angiography
CW: Chicken Wing type
LA: Left atrium
LAA: Left atrial appendage
LAD: Left atrial diameter
LAAFV: Left atrial appendage flow velocity
LVEF: Left ventricular ejection fraction
LVEDD: Left ventricular end diastolic dimension
LVM: Left ventricular mass
LVMI: Left ventricular mass index
IVST: Interventricular septal thickness
LVPWT: Left ventricular posterior wall thickness
TEE: Transesophageal echocardiography
TTE: Transthoracic echocardiography
PaAF: Paroxysmal atrial fibrillation
PeAF: Persistent atrial fibrillation
